# Surface properties of ceria synthesised using Triton-X based reverse microemulsions†

**DOI:** 10.1039/c8ra08947g

**Published:** 2019-03-01

**Authors:** Polyxeni Paschalidou, Charis R. Theocharis

**Affiliations:** Porous Solids Group, Department of Chemistry, University of Cyprus P. O. Box 20537 1678 Nicosia Cyprus charis@ucy.ac.cy

## Abstract

The effect of the tail length of Triton-X surfactants on the surface properties of ceria prepared by means of reversed micelles and Ce(O^i^Pr)_4_ has been systematically studied. Generally, solids with increased surface areas (up to 136 m^2^ g^−1^) were synthesised. It was shown that the tail length strongly affects the surface characteristics. Further studies were carried out using UV-Vis, ATR-FTIR, XRD and TGA/DSC studies of the precursor gels as well as N_2_-isothermal adsorption BET, XRD, FT-IR, UV-Vis diffuse reflectance and SEM investigations of the final solids samples. An interaction mechanism between the ceria precursor molecules and the polar tail of the reversed Triton X micelles and the formation of ceria (CeO_2_) particles in the aqueous nucleus of the reversed microemulsions is proposed.

## Abstract 2

The effect of the tail length of Triton-X surfactants on the surface properties of ceria prepared by means of reversed micelles and Ce(O^i^Pr)_4_ has been systematically studied.

## Graphical abstract

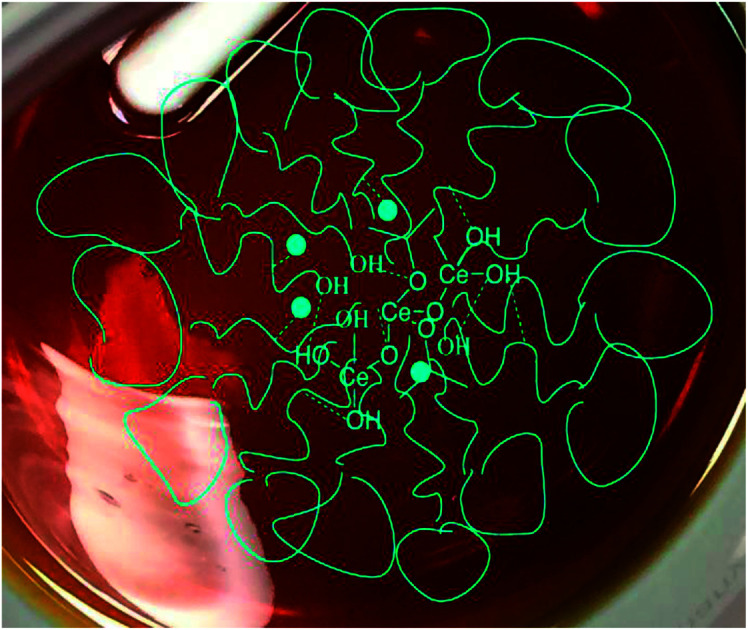

## Introduction

Ceria (CeO_2_) is one of the most reactive metal oxides of the rare earth series and is of particular interest due to a wide spectrum of applications in catalysis, oxygen sensors, solid fuel cells, optoelectronics, environmental pollution or control technologies.^1^ Particularly, mesoporous ceria has attracted considerable attention from the research community and there are several studies dealing with the preparation of mesoporous ceria.^2^ These studies include the use of hydrothermal processes with controlled reaction time^3^ and under high temperature and pressure conditions,^4^ pyrolysis,^5^ and thermal decomposition.^6^ Further, different studies used aqueous precipitation with NH_3_ or NaOH.^7^ Elsewhere, solution and solid combustion methods,^8^ mechanochemical methods,^9^ sol–gel methods,^10–13^ gas phase condensation,^14^ microwave heating^15^ and sonochemical methods were used.^16^

Methods based on reverse microemulsion are of particular interest because they allow particle size and specific surface area control. In addition, using surfactant reverse micelles as microreactors offers control over the particle formation and growth and may affect the hydrolysis rate of the precursor compound.^17^ Moreover, the solid synthesis in reverse micelle microreactors results in the formation of almost monodispersed particles.^18^ The ability of surfactants to cover the formed particles often favors the formation of specific crystal phases, determines particle shape and surface properties, and prevents particle agglomeration.^17^

Ceria can be used in a variety of catalytic applications, in oxygen sensors, fuel cells, UV radiation absorbers, in antipollution technology, among others. The usefulness of the material arises from two factors. First, the thermal stability of the fluorite structure allowing the uptake or release of oxygen from the lattice due to Ce(iii) to Ce(iv) conversion and *vice versa*. Second, the optoelectronic properties of the solid and how they can be controlled *via* changes to the band structure. Both these parameters are influenced by particle size and pore structure.

There are no systematic studies related to ceria synthesis using water in oil microemulsions and particularly employing neutral surfactants (*e.g.* Triton-X) of varying polar tail length and Ce(O^i^Pr)_4_ as precursor. Moreover, there are no investigations addressing the interaction between the ceria precursors and the Triton-X surfactants as well as the CeO_2_ crystallite formation within the aqueous nucleous of the reversed micelles. Hence, the aim of the present study is the preparation of ceria nanoparticles using reversed micelles of Triton-X with varying tail length and Ce(O^i^Pr)_4_, and following investigation of their surface characteristics after thermal pre-treatment at 300 °C or direct calcination at different temperatures (*e.g.* 400 °C, 500 °C and 600 °C).

The aim of the present study is to use micelles as a medium for the synthesis of nanoporous ceria with well-characterized pore structure and surface chemistry, with the ultimate aim of using these solids as catalysts on the conversion of nitrogen oxides to dinitrogen. The study is part of a series of investigations carried out at the University of Cyprus of studying the synthesis of well-ordered nanoporous solids.

## Experimental

### Ce(O^i^Pr)_4_ preparation

Ce(O^i^Pr)_4_ has been synthesized by mixing the freshly prepared sodium isopropoxide (NaO^i^Pr) and the dried ammonium cerium(iv) nitrate (extra pure, HiMedia) in DME (1,2-dimethoxyethane). NaO^i^Pr was prepared by reacting sodium flakes (Fisher Chemical, laboratory reagent grade) with excess of isopropanol (99.95% ^i^PrOH, Fisher Chemical) in DME (≥99%, Sigma-Aldrich). The mixture was stirred overnight under N_2_ atmosphere and the resulting reddish solution, which contained the product (Ce(O^i^Pr)_4_) was separated from the precipitate (NaNO_3_) by vacuum filtration under an inert N_2_ atmosphere. Following this step, the filtrate was evaporated to 10 percent its initial volume and used as it was for the ceria synthesis.^19,20^ The chemical structure and properties of the concentrate were identified and characterized by ATR-FTIR^19–24^ and ^1^H & ^13^C NMR spectroscopy.^25^

### Reversed micelle-mediated ceria synthesis

The ceria gels have been prepared by mixing the Ce(O^i^Pr)_4_ concentrate with the three different reversed micelle mixtures of Triton X-100 (extra pure, Fisher Chemical), Triton X-114 (extra pure, Sigma-Aldrich) and Triton X-45 (extra pure, Sigma-Aldrich) at a Ce/H_2_O-molar ratio 1 : 1.^10^ The different micelle mixtures were prepared by mixing the corresponding Triton-X surfactant and de-ionized water (molar ratio (Triton-X/H_2_O) = 0.8) in cyclohexane (99.98%, Fisher Chemical) at a concentration of 1.2 mol kg^−1^. Then, the concentrated precursor (Ce(O^i^Pr)_4_) solution was added to the reverse micelles mixtures. After two days and under ambient conditions the sols were transformed to clear gels and aliquots of these gels have been calcined at different temperatures (300 °C, 400 °C, 500 °C and 600 °C) for two hours at a temperature rate of 6 °C min^−1^. For comparison, aliquots of the 300 °C samples were also calcined at 400 °C, 500 °C and 600 °C at a temperature rate of 6 °C min^−1^.

### Physicochemical characterization of gels and powders

The precursor compound, Ce(O^i^Pr)_4_, the gels and the powders resulted after calcination were characterized using a variety of methods.

^1^H- and ^13^C-NMR spectra were recorded on a Bruker Avance 300 instrument (at 300 and 75 MHz, respectively). ^13^C-DEPT NMR was used to identify quaternary and tertiary carbons. Deuterated solvents were used for homonuclear lock and the signals are referenced to the deuterated solvent peaks.

The measurements of UV-Vis absorption spectrophotometry, isothermal N_2_ adsorption/desorption (employing a slit-shaped pore model), powder X-ray diffraction spectroscopy, Fourier-Transform Infrared spectrophotometry, ATR-FTIR (attenuated total reflectance) Fourier-transform infrared spectroscopy, UV-Vis diffuse reflectance solid state spectroscopy, thermogravimetric analysis, thermal differential scanning calorimetry and scanning electron microscopy were carried out as described elsewhere.^12^

## Results and discussion

### Ceria gels

**Fig. 1:**
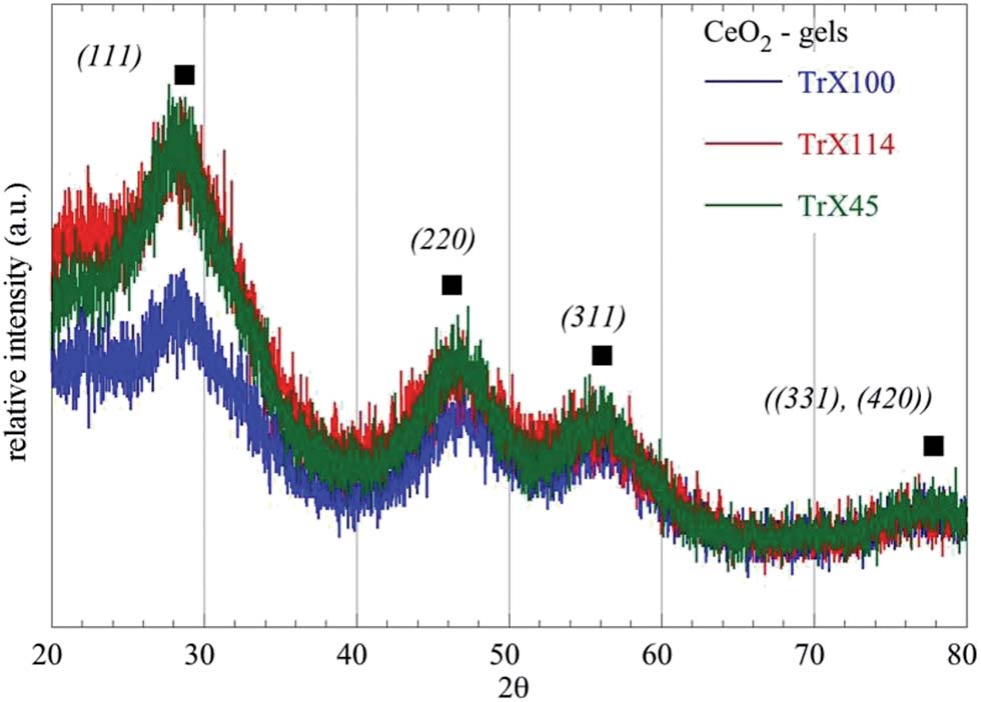
X-Ray diffractograms of the ceria gels obtained using three different Triton-X surfactants.

The prepared ceria gels using the three different Triton-X surfactants (Triton X-100, Triton X-114 and Triton X-45) are clear and have light brown color indicating their successful preparation. According to the XRD data (Fig. 1 and Table 1) of the gels increasing the polar tail length of the surfactant results in the formation of larger ceria particles. As the polar tail length of the surfactant increases stereochemical hindrances and repulsions between the polar tails are favored and the aqueous core diameter of the reversed micelles increases.^17,26^ Space availability within the aqueous core allows the formation of larger particles, whereas when the space is limited the formation of smaller particles is favored.^27,28^

**Table 1:** XRD data of the three different ceria gels corresponding to Triton X-100, Triton X-114 and Triton X-45

Sample – CeO_2_	2*θ*_Bragg_ (°)	Level	*d*_Bragg_ (Å)	FWHM (°)	*L*_XRD_ (nm)
Gel TrX-100	27.87	(111)	3.0131(10)	1.8	4.75(2)
Gel TrX-114	27.41	(111)	3.2516(10)	2.27	3.77(2)
Gel TrX-45	26.95	(111)	3.306(10)	2.48	3.44(2)

This observation was corroborated also by the UV-Vis absorption spectra of the gels as described in Table 2. Specifically, the energy gap for the ceria particles, which indicates that the ceria particle size within the gels decreases with decreasing of the polar tail of surfactant length (TrX-100 (*n*_ethoxy_ = ∼9.5) > TrX-114 (*n*_ethoxy_ = ∼7.5) > TrX-45 (*n*_ethoxy_ = ∼4.5)).

**Table 2:** UV-Vis absorption data of the three different ceria gels corresponding to Triton X-100, Triton X-114 and Triton X-45

Sample	CeO_2_ peak (nm)	*para*-Disub. phenol group (nm)	*para*-Disub. phenol group (nm)	Energy gap CeO_2_ (eV)	*L*_XRD_ (nm)
Gel TrX-100	229.1	277.4	284.2	5.42	4.75
Gel TrX-114	225.6	277.4	284.2	5.5	3.77
Gel TrX-45	224.4	277.4	284.2	5.53	3.44

### Surface properties of the CeO_2_ solids

**Fig. 2:**
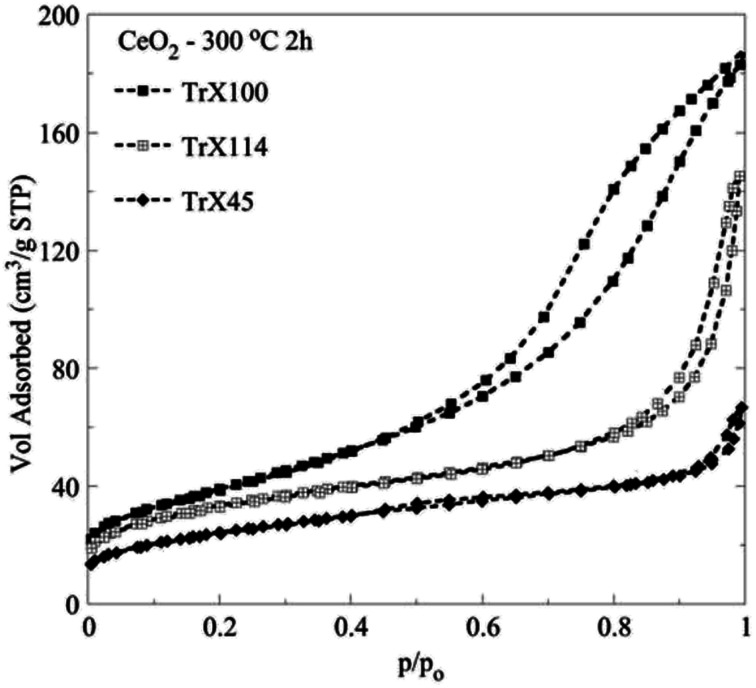
Adsorption/desorption N_2_ isotherms of the ceria solids prepared by means of three different Triton-X surfactants after calcination at 300 °C for 2 h.

Fig. 2 shows the N_2_ adsorption/desorption isotherms of the CeO_2_ solids prepared after thermal treatment of the corresponding gels at 300 °C for 2 h. According to IUPAC, the isotherms are type II with a H3 hysteresis loop^29,30^ and are characteristic for pseudomesoporous materials with irregular pores of slit type geometry. Moreover, the corresponding data summarized in Table 3 show that there is a linear dependence between the specific surface area of the ceria solids and the tail length of the surfactant used. However, there is no clear correlation between the average pore size and the particle size, because the pores are formed during the thermal treatment of the gels, and is a complex process. Moreover, these pores are caused by the aggregation of primary and secondary particles, and not within those. In that latter case, there would had been a strong correlation between particle size as measured by XRD and pore size. Nevertheless, the ceria solid corresponding to the Triton X-100 with the longest tail length presents the smallest average pore diameter and the highest specific area and pore volume.^26,30^ Taking into account that in the gels the particle size was directly proportional to polar tail length of the surfactants, it is evident that the surfactant affects particle aggregation and porosity during thermal pre-treatment of the gels and removal of the organic matrix.

**Table 3:** Surface properties of the ceria solids prepared by means of three different Triton-X surfactants after calcination at 300 °C for 2 h evaluated from the N_2_ adsorption/desorption isotherms

Sample	*S*_BET_ (m^2^ g^−1^)	*V*_Sp_ (cm^3^ g^−1^)	*d*_BJH_ (nm)	*d*_DFT_ (nm)
CeO_2_ TrX-100 (*n* = ∼9.5) 300 °C 2h	134.8	0.282	6.53	9.16
CeO_2_ TrX-114 (*n* = ∼7.5) 300 °C 2h	117.4	0.199	10.28	9.3
CeO_2_ TrX-45 (*n* = ∼4.5) 300 °C 2h	84.4	0.083	5.44	3.85

**Fig. 3:**
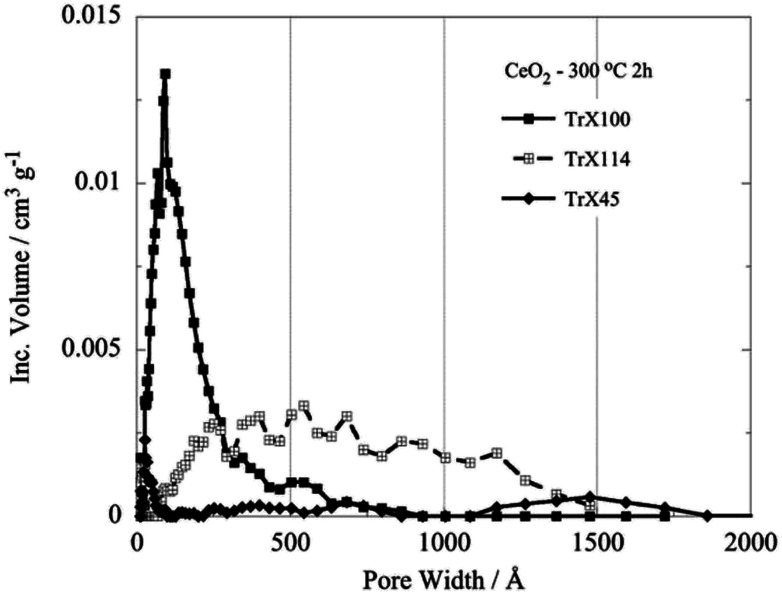
DFT pore size distributions of the ceria solids corresponding to the three different Triton-X surfactants after calcination at 300 °C for 2 h.

Similarly, decreasing length of the surfactant polar tail results in declining in the size of the hysteresis loop and hence decreasing of the specific pore volume and BJH pore diameter.^31^ There is almost linear correlation between the pore volume and the size of the surfactant tail length. The corresponding values decrease linearly with the number of the ethylenoxy groups of the hydrophilic tail of Triton-X. Fig. 3 shows the pore size distribution, which has been obtained after evaluation of the N_2_ adsorption/desorption isotherm data by a DFT method^32^ and correspond to the three different Triton-X surfactants (Triton X-100, Triton X-114 and Triton X-45) after calcination at 300 °C for 2 h. According to Fig. 3 the pore size distribution for Triton X-100 is relatively narrow and is restricted in the mesoporous area between 2 and 50 nm, whereas the ceria solids corresponding to the other two surfactants present a much wider pore size distribution. Moreover, Fig. 3 clearly shows that the pore volume decreases with decreasing the surfactant tail length.

Characterisation of the ceria solids by XRD (Table 4) indicates that all solids have a pure face centered cubic lattice and *Fm*3*m* space group.^33^ Moreover, the data corroborate the surface analysis data (Fig. 2 and Table 3) because after calcination the particle size of the solid obtained from the surfactant with the largest polar tail (*e.g.* Triton X-100) has the average smallest particle assuming that the smallest particles are associated with the highest specific area and pore volume.^26,30^ Comparing the ceria particle size in the gels and in the solids (*e.g.* after thermal treatment at 300 °C for 2 h) it is clear that the particle size of the ceria particles is increased from 4.75 nm to 6.58 nm in the case of Triton X-100. Further, particle size is almost doubled in the case of the other two surfactants, indicating the role of the polar tail length in the agglomeration/crystallisation process during calcination and matrix removal.

**Table 4:** XRD data of the ceria solids corresponding to the three different Triton-X surfactants after calcination at 300 °C for 2 h

Sample	*S*_BET_ (m^2^ g^−1^)	2*θ*_Bragg_ (Å)	Level	*d*_Bragg_ (Å)	FWHM (°)	*L*_XRD_ (nm)
CeO_2_ TrX-100 300 °C 2h	134.8	28.65	(111)	3.1134(10)	1.3025	6.58(2)
CeO_2_ TrX-114 300 °C 2h	117.4	28.49	(111)	3.1309(10)	1.2105	7.08(2)
CeO_2_ TrX-45 300 °C 2h	84.4	28.45	(111)	3.1347(10)	1.22	7.02(2)

**Fig. 4:**
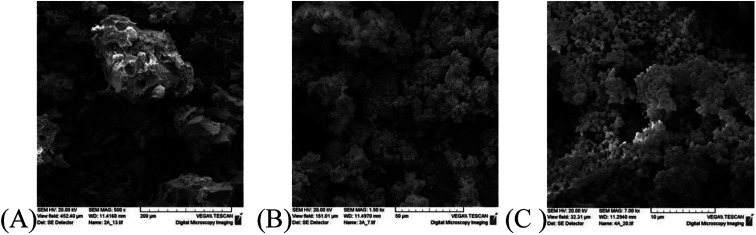
SEM pictures of the ceria solids corresponding to the three different Triton-X surfactants after calcination at 300 °C for 2 h.

However, the difference in the crystal size between the solids obtained from Triton X-114 and Triton X-45 is insignificant indicating complex interactions between the ceria precursors and the polar surfactant tails. Furthermore, it becomes evident that not only the crystallite size but also their agglomeration and particle morphology determine the porosity of the solids. The morphology of the three different solids as determined by SEM is shown in Fig. 4 and indicates that the morphologies of the solids prepared using Triton X-114 and Triton X-45 are closer to one another.

### The effect of calcination temperature on the surface properties of the CeO_2_ solids

**Fig. 5:**
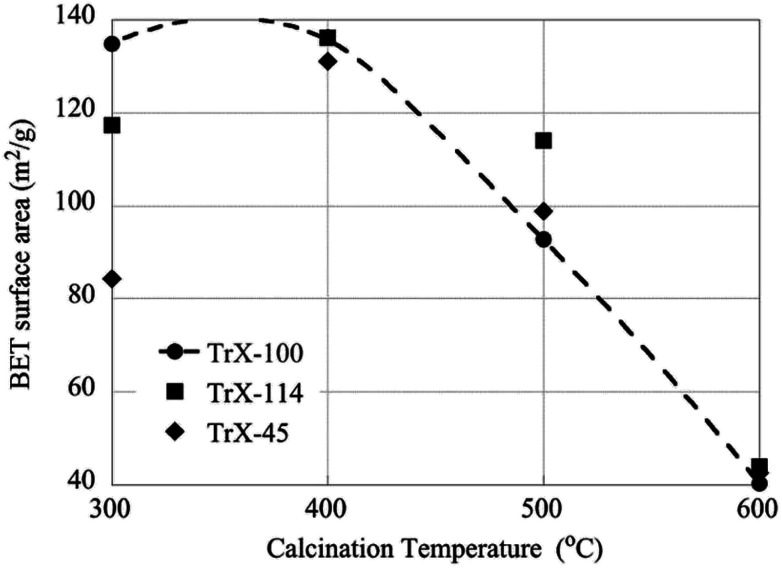
The effect of calcination temperatures on the BET surface area of the CeO_2_ solids.

The effect of higher calcination temperatures on the BET surface area is graphically summarized in Fig. 5. According to Fig. 5 gel treatment at 400 °C results generally in higher surface areas which could be attributed to pore formation due to thermal-induced re-arrangement of the crystallites or also because of decomposition of entrapped organic phase. It is noteworthy, that the effect is more prevalent with decreasing the length of the surfactant tail. However, above 400 °C the surface area decreases linearly with temperature and reaches a value of about 40 m^2^ g^−1^ independent of the Triton-X surfactant. Moreover, calcination results in dramatic changes in the pore size distribution as indicated DFT analysis, which shows a widening of the pore size distribution of the solid associated with Triton X-100 and pseudo-pore formation and increase in porosity of the solids corresponding to Triton X-114 and Triton X-45.

**Fig. 6:**
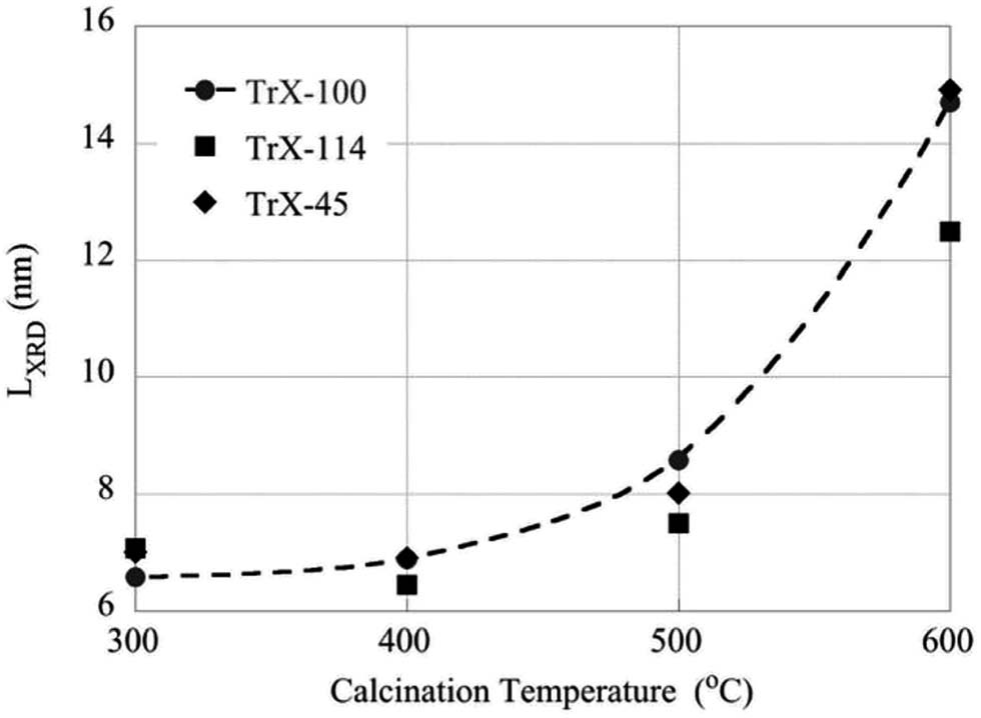
The effect of calcination temperatures on the crystallite size of the CeO_2_ solids as determined by XRD measurements.

On the other hand the crystallite size of the ceria solids as evaluated from the XRD data (Fig. 6) increases with increasing calcination temperature due to fusion of smaller crystallites to bigger ones. Interestingly, increasing calcination temperature has less effect on the crystallite growth of the solids corresponding to surfactants with smaller tail and particularly the Triton X-114, which is in agreement with the changes in surface area. Moreover, this behavior coincides with the corresponding behavior of TiO_2_ solids indicating on similar interaction mechanisms.^12^

**Fig. 7:**
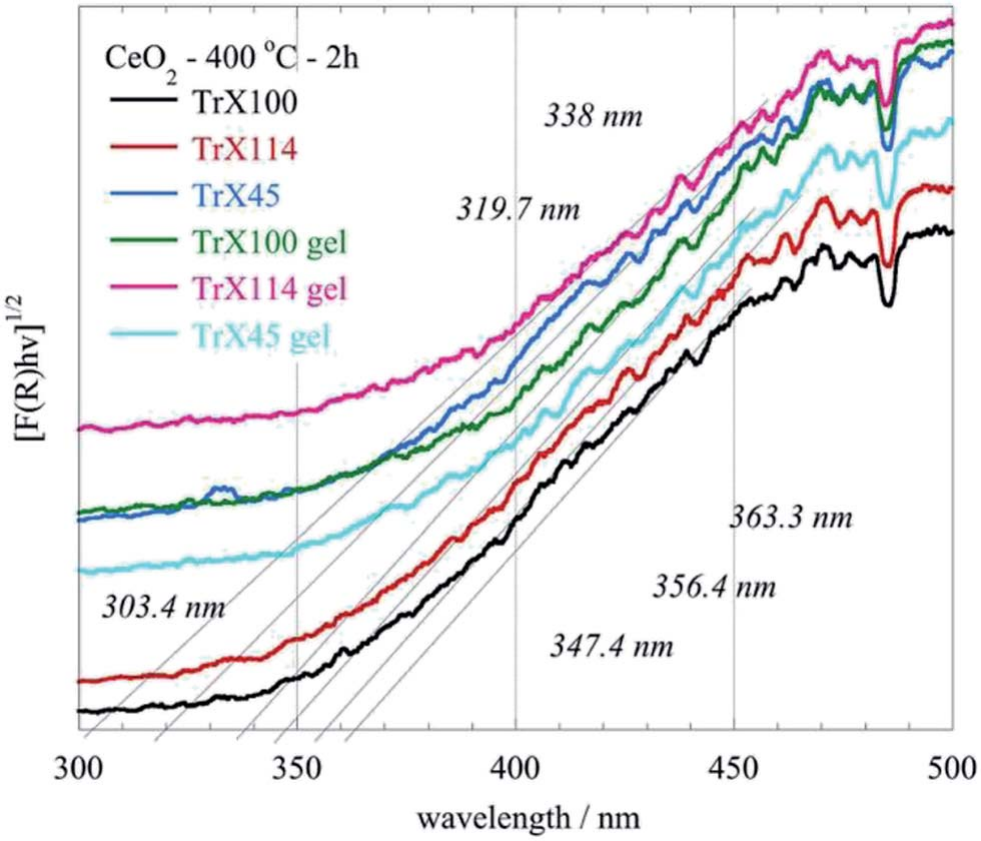
DRUV-Vis spectra of the CeO_2_ solids obtained after direct (gel) and indirect calcination at 400 °C for 2 h of the three different Triton-X reversed micelles gels.

The XRD and BET surface data are corroborated by the DRUV-Vis data (Fig. 7) corresponding to ceria solids obtained after direct and indirect calcination at 400 °C for 2 h of the three different Triton-X gels. In these spectra the absorbance *F*(*R*) has been evaluated from the reflectance using the Kubelka–Munk model.^34^ According to Fig. 7 the ceria particles obtained by direct calcination of the gels present significantly higher energy gap due to their smaller size. Moreover, the energy gap decreases with increasing the length of the hydrophilic tail because the particle size is increasing. The latter is associated with the radius of the aqueous center of the reversed micelles.

### FTIR studies of the CeO_2_ samples

**Fig. 8:**
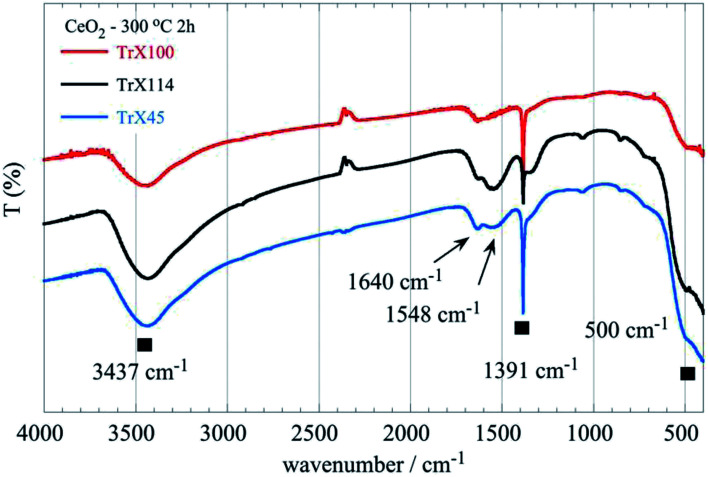
FTIR spectra of the ceria solids obtained after calcination of the corresponding Triton X-100, 114 and 45 reversed micelles at 300 °C for 2 h.

The FTIR spectra of the ceria solids obtained after calcination of the corresponding Triton X-100, 114 and 45 reversed micelles at 300 °C for 2 h are shown in Fig. 8. The associated data are summarized in Table S4 (ESI†). These include the characteristic groups and their maxima in the spectra as well as relevant literature data.^2,33,35–42^

All spectra show a characteristic sharp peak at 1391 cm^−1^ which is characteristic^41,42^ for CeO_2_ but could be also attributed to the stretching mode of COO and Ce–O–C^36,37^ or ⋯Ce–O–H.^43^ The Ce–O–C bonds were, presumably, formed when organic residues trapped within the pores interact with the ceria surface. Furthermore, when the length of the polar tail of the Triton-X surfactant increases the strength and the width of the broad band between 640 and 400 cm^−1^ decreases gradually, indicating differences in the Ce–O bond length between the solids obtained from the three different Triton-X reversed micelles.

### Possible mechanism of the Triton X-100 reversed micelle-mediated ceria particles formation

**Fig. 9:**
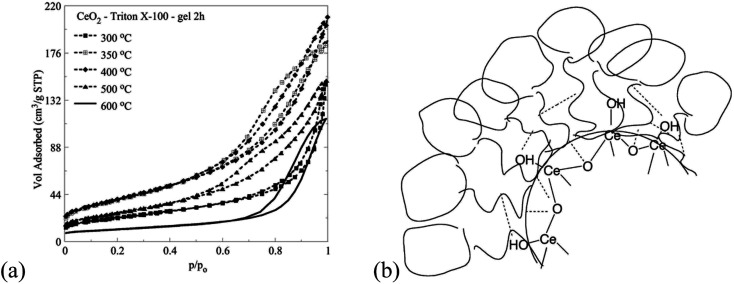
N_2_ ad/desorption isotherms of ceria particles corresponding to Triton X-100 after direct calcination at different temperatures (a) and schematic illustration of possible interactions between the Triton X-100 polar tails and the ceria precursors (b).

The interaction of the Triton X-100 surfactants with the inorganic ceria precursors favours the formation of mesoporous ceria particles.^10,44^ The associated N_2_ ad/desorption isotherms are summarized in Fig. 9. According to IUPAC the isotherms from type II with a H3 hysteresis loop transformed to type IV (a) with a H2 (b) hysteresis loop.^45^ This occurs because the ceria nano-crystalline particles in the center of the reversed micelles are covered by the Triton X-100 molecules due to their large free surface energy. The coverage by the Triton X-100 surfactant tail affects the pore formation, the structure and texture of the formed ceria particles.^26,44^

The synthetic route for ceria that is discussed in this paper provides a better control of the formation of the pore structure of the product, *via* two routes. The first is the use of sol–gel conditions which result in a slow precipitation of the ceria solid than obtains in the homogeneous route, and the second is the separation of the metallo-organic ceria precursor from the aqueous phase by the micelles. As polymerization of the –Ce–O–Ce– bonds takes place, crystallites and then primary particles precipitate out within the confined aqueous core of the micelle. However, the confined space will not allow the development of intraparticle pores, nor the formation of larger aggregates. The precipitated inorganic solid causes a collapse of the micelles, allowing the particles therein to aggregate and precipitate out within the supernatant liquid, resulting in interparticle pores. The formation of aggregates is controlled by the extent of covering of the surface of the growing particles by Triton-X moieties, the presence of which influences the size and porosity of the aggregates and thus surface area, pore volume, *etc.* Clearly, different chain size will result in a different mode of adsorption and thus different effect on the precipitate. The synthesis described herein gives consistent and repetitive results.

## Conclusions

The following conclusions can be gleaned from the present study:

• The specific surface area and volume of the solids CeO_2_ decrease linearly with decreasing the length of the hydrophilic surfactant tail. The effect is exactly opposite regarding the crystallite size.

• After calcination at 400 °C the specific surface area increases particularly for the ceria solids corresponding to Triton X-114 and Triton X-45 surfactants. For calcination temperatures above 400 °C the specific surface area decreases almost linearly with temperature and reaches a value of ∼40 m^2^ g^−1^ independent of the ceria solid. On the other hand, the crystallite size decreases exponentially due to fusion of smaller particles.

• After calcination at 400 °C the solid associated with the Triton X-114 surfactant presents the highest specific surface area similar to TiO_2_ prepared by means of reversed micelles of Triton-X, indicating similar interaction mechanisms.

• The N_2_ ad/desorption isotherms, XRD and DRUV-Vis data indicate that direct calcination of the ceria gels results in the formation of ceria solids with superior surface properties.

• At increased calcination temperatures pseudo-mesoporous (slit-type) ceria samples are transformed to solids with well-defined mesopores. The surface chemistry of the solids compares well with those prepared and described elsewhere.^46,47^

## Conflicts of interest

There are no conflicts to declare.

## Supplementary Material

RA-009-C8RA08947G-s001
