# Supplementary material for: Surface properties of ceria synthesised using Triton-X based reverse microemulsions
Source: RSC Adv. 2019 Mar 1;9(12):7025–31. doi: 10.1039/c8ra08947g (PMC9061172; doi:10.1039/c8ra08947g)
Supplement: RA-009-C8RA08947G-s001 [file RA-009-C8RA08947G-s001.pdf]

Electronic Supplementary Material (ESI) for RSC Advances.
This journal is © The Royal Society of Chemistry 2019

Supplementary information

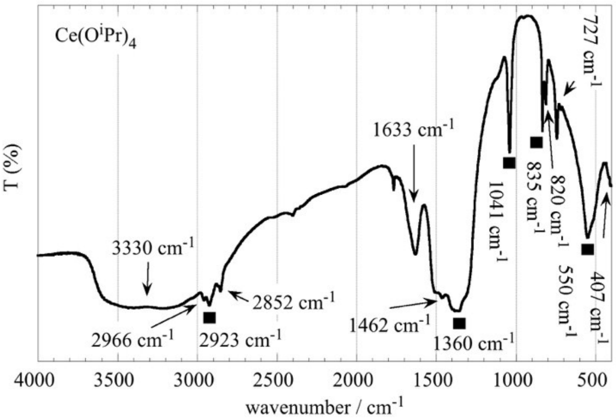

Figure S1: *FTIR-ATR spectrum of the $Ce(O^iPr)_4$ concentrate in the range 4000 - 400 cm^{-1}.*

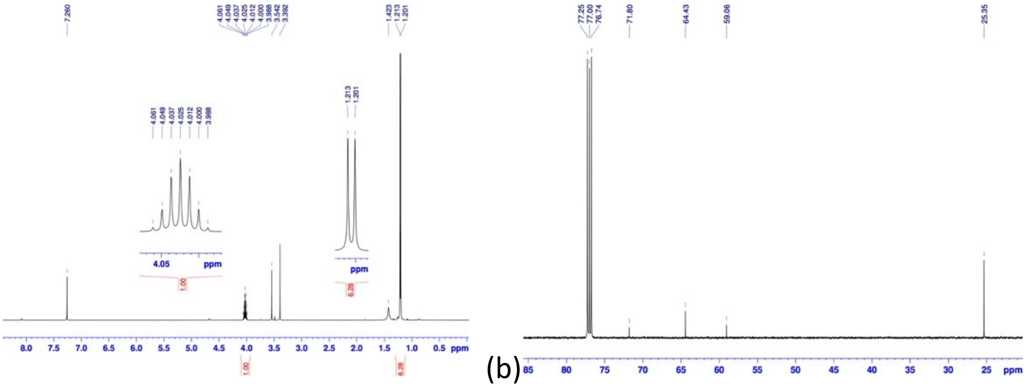

(a) (b)

Figure S2: *^1H (a) & ^{13}C (b) NMR spectra of the cerium(IV) isopropoxide concentrate.*

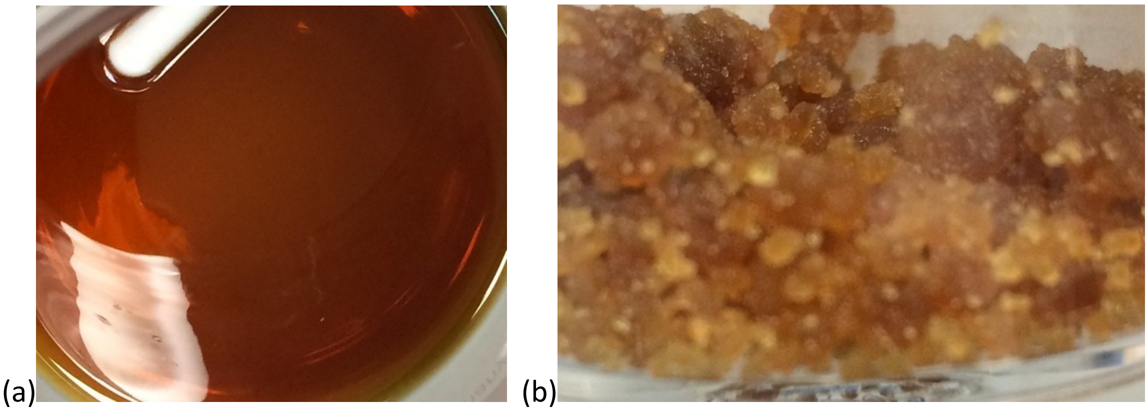

(a) (b)

Figure S3: *Images of the ceria gel (a) corresponding to Triton X-100 and (b) the gel which has been obtained when the precursor compound $Ce(O^iPr)_4$ was hydrolyzed outside of the aqueous cores of the reverse micelles.*

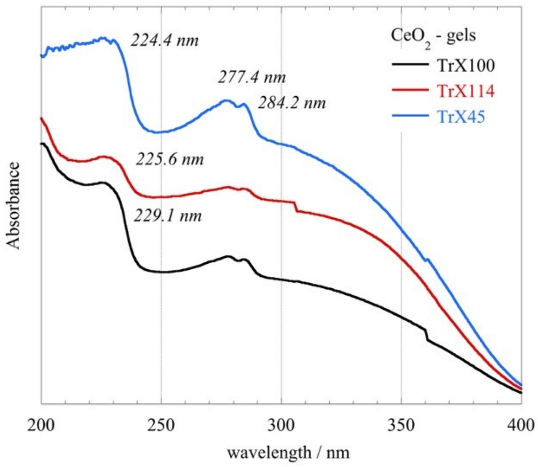

Figure S4: *UV-Vis Absorption spectra of the three different ceria gels corresponding to Triton X-100, Triton X-114 and Triton X-45.*

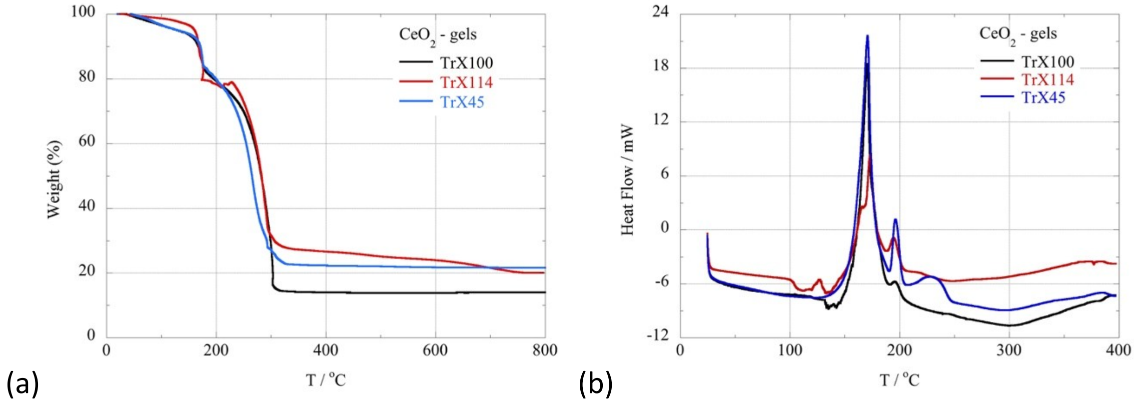

Figure S5: *(a) TGA and (b) DSC profiles of the three different ceria gels corresponding to Triton X-100, Triton X-114 and Triton X-45 reverse micelles.*

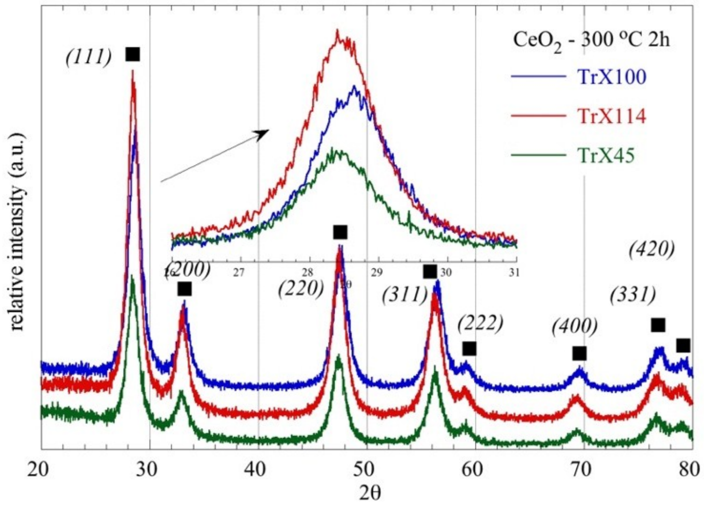

Figure S6: *X-Ray Diffractograms of the ceria solids corresponding to the three different Triton-X reverse micelles after calcination at 300 °C for 2 h.*

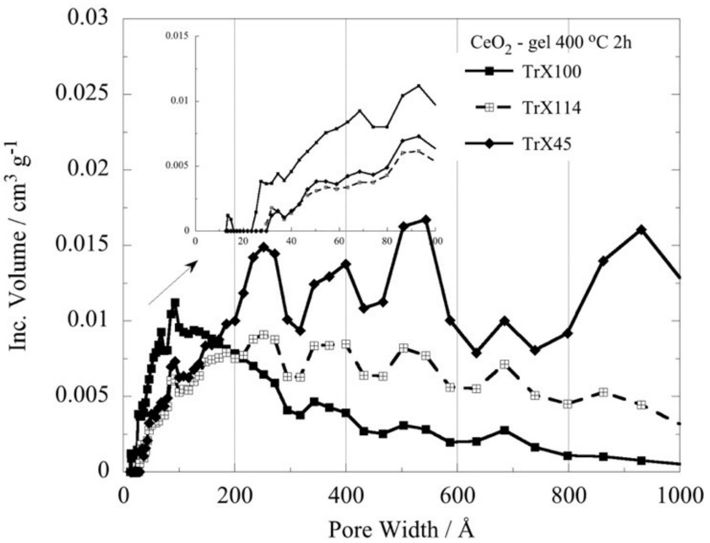

Figure S7: *DFT pore size distributions of the ceria solids obtained from the three different Triton-X surfactants gels after calcination at 400 °C for 2 h.*

Table S1: *FTIR-ATR data of the Ce(O^{i}Pr)$_4$ precursor compound.*[19-24]

functional group	bibliographical characteristic absorption (cm^{-1})	experimental characteristic absorption (cm^{-1})
stretching vibration of hydroxyl groups	3500	3330
-C-H elongation vibration	2968	2966
anti-symmetric stretching vibration of -C-H	2930	2923
symmetric stretching vibration of -C-H	2868	2852
bending vibration of -OH$^-$	1600	1633
bending vibration of -C-H	1480	1462
CH_3-C-CH_3 stretching modes of the isopropoxy group	1350	1360
stretching conjugated vibration of -C-O	1050	1041
C-C stretching vibration within the isopropoxy group	< 1000	835
skeletal vibration of the isopropoxy group	841	820
symmetrical skeletal vibration of the isopropoxy group	785	727
skeletal vibration of the isopropoxy group	566	550
stretching vibration of Ce-OR	406	407

Table S2: *^{1}H και ^{13}C NMR (ppm) experimental data of Ce(O^{i}Pr)$_4$ in CDCl$_3$ solvent.*[25]

^{1}H NMR		^{13}C NMR	
δ (ppm)	compound	δ (ppm)	compound
(1.201 & 1.213) doublet, 24 H	isopropoxide (CH_3)	25.35, 8 C	isopropoxide (CH_3)
1.423 single	water (OH)	59.06	DME (CH_3)
3.392 single	DME (CH_3)	64.43, 4 C	isopropoxide (CH)
3.542 single	DME (CH_2)	71.8	DME (CH_2)
(3.988, 4, 4.012, 4.025, 4.037, 4.049 & 4.061) septet, 4 H	isopropoxide (CH)	(76.74, 77 & 77.25)	solvent ($CDCl_3$) signals
7.26	solvent ($CDCl_3$) residual signals		

Table S3: *TGA and DSC data of the three different ceria gels corresponding to Triton X-100, Triton X-114 and Triton X-45 reverse microemulsions.*

TGA - sample - CeO_2	temperature range (°C)	weight loss (%)*
gel Triton X-100	34.1 - 143.3<br>145 - 187.6<br>192.8 - 320.7<br>324.1 - 385.5	7.58<br>17.96<br>91.02<br>1.2
gel Triton X-114	30.7 - 127.9<br>131.3 - 179.1<br>182.5 - 325.8<br>329.2 - 586.8<br>598.7 - 765.9	2.7<br>23.89<br>68.98<br>5.01<br>5.4
gel Triton X-45	40.9 - 131.3<br>133.1 - 179.1<br>179.1 - 327.5<br>358.2 - 557.8	5.05<br>11.3<br>62.44<br>1.19

** The weight loss values are normalized.*

DSC - sample - CeO_2	temperature range (°C)	max peak temperature (°C)	enthalpy (J/g)	combustion total enthalpy (J/g)
gel Triton X-100	130.9 - 140.5<br>142.9 - 190.6<br>191.8 - 206.7<br>265.1 - 333.1	133.2<br>170.5<br>196.4<br>302.3	endo: 2.7<br>exo: 326<br>exo: 5.4<br>endo: 32.4	exo: 414.8
gel Triton X-114	97.5 - 127.4<br>135.1 - 187.6<br>188.8 - 206.1<br>215.6 - 274.7<br>318.8 - 392.7	111.7<br>173<br>195.2<br>243.2<br>363.1	endo: 22.5<br>exo: 174.9<br>exo: 15.9<br>endo: 20<br>exo: 25.4	exo: 304.9
gel Triton X-45	129.7 - 189.4<br>190.6 - 206.1<br>208.5 - 247.8<br>256.8 - 341.4<br>357 - 393.3	170.8<br>196.3<br>231.1<br>297.9<br>383.5	exo: 389.4<br>exo: 44.4<br>exo: 48.4<br>endo: 47<br>exo: 7.6	exo: 515.3

Table S4: *Experimental and literature FTIR data for the CeO_2 solids obtained from the three different Triton-X reversed micelles.*[2,33,35-42]

functional group	bibliographical characteristic absorption (cm^{-1})	experimental characteristic absorption (cm^{-1})
stretching vibration of physisorbed H_2O or OH^- stretching frequency of unidentate Ce-OH	3440	3437
asymmetric stretch of CO_2	2350	2353
stretching vibration of the hydrogen bonded C=O group	1783	1769
bending vibration of $-OH^-$	1620	1640
asymmetric stretching vibration of C-O-C at para-disub phenol or asymmetric stretching vibration of the $RCOO^-$	1540	1548
stretching vibration of COO & Ce-O-C or Ce-O-Ce	1380	1391
stretching vibration of Ce-O	850	835
stretching vibration of the Ce-O-Ce	710	671
stretching vibration of the Ce-O	500	500
δ(Ce-O...O) stretching mode of vibration	534, 526 & 497	500-400
